# The complete mitochondrial genome of *Trematomus loennbergii* (Perciformes, Nototheniidae)

**DOI:** 10.1080/23802359.2021.1899070

**Published:** 2021-03-18

**Authors:** Eunkyung Choi, Tae-Eul Im, Seung Jae Lee, Euna Jo, Jinmu Kim, Sun Hee Kim, Young Min Chi, Jeong-Hoon Kim, Hyun Park

**Affiliations:** a College of Life Sciences and Biotechnology, Korea University, Seoul, Korea; b Greenwitch Co, Chungcheongbuk-do, Korea; c Division of Polar Life Science, Korea Polar Research Institute, Incheon, Korea

**Keywords:** Mitochondrial genome, *Trematomus loennbergii*, Scaly rockcod, PacBio

## Abstract

The complete mitochondrial genome of *Trematomus loennbergii* was studied using NGS technology with PacBio platform. The mitochondrial genome size was 19,374bp and it had 13 protein-coding genes, 22 tRNAs and 2 rRNAs. There were 4 types of stop codons which were TAA, TAG, AGG and T(AA) but start codon type was only one (ATG). The contents of GC were 44.09% and AT contents were 55.91%. To conduct phylogenetic analysis, 12 species in 3 families were used. The result suggested that *T. loennbergii* was close to *Pagothenia borchgrevinki* in Nototheniidae. This study would provide a fundamental data for molecular evolution of *T. loennbergii.*

The Nototheniidae family of Antarctic fishes have various characters, for example, antifreeze glycoprotein (AFGP) and lack of hemoglobin and these are different with other teleost fishes (Clarke and Johnston 1996 and DeVries and Cheng 2005). The subfamily Trematominae has only 14 species and among these Trematomus is main genus having 11 species and their habit distribution is very wide (Lautredou et al. 2010). In addition, the morphology of Trematomus species is very similar, for example, *Trematomus loennbergii* and *Trematomus lepidorhinus* are only different in absence or presence of scales on the preorbital and lower jaw (DeWitt et al. 1993).

*Trematomus loennbergii* (Regan, 1913) is known as Scaly rockcod and their distribution is Southern Ocean such as coasts of Antarctic Peninsula, Queen Mary, Adelie, South Victoria Coasts, Davis, Ross and Weddell seas according to fish base (https://www.fishbase.se/summary/7057). Their depth distribution is usually 65–832 m and common length is 20 cm. They feed on a wide range of prey and main food resources are epifaunal and tube-dwelling polychaetes (Mesa et al. 1997).

The biological information of *Trematomus loennbergii* has not been studied well and the completed mitochondrial genome information is not available so far. In this study, we conducted the first full-length mitochondrial genome assembly of *Trematomus loennbergii* with the next generation sequencing (NGS) technology, PacBio platform. We also analyzed phylogenetic tree to know the evolutionary position in Antarctic fish families.

*Trematomus loennbergii* was collected from Ross Sea (77°05’S, 170°30’E on CCAMLR Subarea 88.1), Antarctica and then transferred to −80 °C freezer in laboratory. The specimen was deposited at the Earth Biocollection in the Division of Biotechnology, Korea University (http://genome.kusglab.org/collections.html, amy_choi@korea.ac.kr) under the voucher number KAN0003030. The DNA was extracted from frozen muscle tissue by the conventional phenol-chloroform method. For the quality and quantity check, Fragment analyzer (Agilent Technologies, CA, USA) and Qubit 2.0 Fluorometer (Life Technologies, CA, USA) were used respectively. To conduct sequencing step, the 20 kb fragmentation was done using Covaris G-tube (Covaris, MA, USA) and the SMRTbell library was constructed using SMRTbell™ Template Prep Kit 1.0 according to manufacturer’s protocol. Sequel Binding Kit 3.0 and SMRTbell Template Prep Kit 1.0 were used to construct SMRTbell-Polymerase and SMRTbell library Complex. After this step, the complex was load into the Sequel platform using Sequel Sequencing Kit 3.0 and SMRT cells 1 M v3. Total 4 SMRT cells were used to perform sequencing step in the Sequel sequencing platform (Pacific Biosciences, CA, USA). Subreads of PacBio for mitochondria genome assembly were filtered out using 16 s rRNA and COI gene sequences and then CANU assemble 1.8 (Koren et al. 2017) was used for *de novo* mitochondrial genome assembly. The assembled sequences were retrieved into MITOS (Bernt et al. 2013) web service for mitochondrial genome annotation.

The complete mitochondrial genome size of *T. loennbergii* (GenBank Number: MT447073) was 19,374bp and it consisted of 13 protein-coding genes, 2 rRNAs and 22 tRNAs. Start codon was only ATG in 13 protein coding genes but there were 4 types of stop codon. 6 protein coding genes (ND3, ND2, COX1, ATP6, COX3 and ND4L) had TAA stop codon, 3 protein coding genes (ATP8, ND3 and ND5) had TAG stop codon, 3 protein coding genes (COX2, ND4 and Cytb) had T(AA) stop codon and only ND6 had AGG as a stop codon. The contents of GC were 44.09% and AT contents were 55.91%.

A phylogenetic tree (Figure 1) was analyzed to know the evolutionary position of *T. loennbergii*. The MEGA X (Kumar et al. 2018) by Maximum Likelihood method with 1,000 bootstrap replications and JTT matrix-based model (Jones et al. 1992) were used for this analysis. As a result, the data showed that *T. loennbergii* was clustered with *Pagothenia borchgrevinki* in Nototheniidae family. This study would provide the fundamental data to understand the evolutionary relationship with other fishes in Antarctic region.

**Figure 1.**
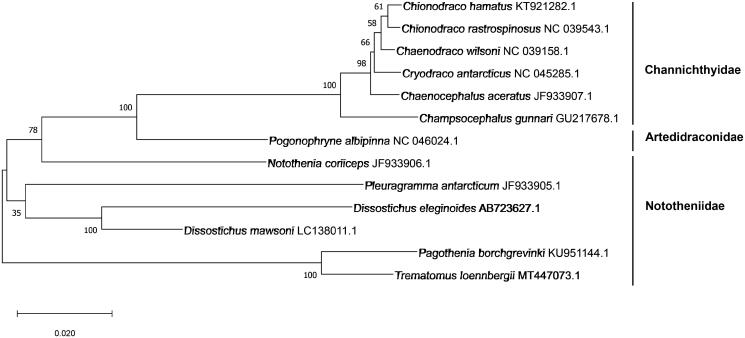
The phylogenetic tree using complete mtDNA sequence with MEGA X by Maximum Likelihood method and JTT matrix-based. The GenBank number and scientific name were included for each species.

## Data Availability

The genome sequence data that support the findings of this study are openly available in GenBank of NCBI at (https://www.ncbi.nlm.nih.gov/) under the accession no. MT447073. The associated BioProject, SRA, and Bio-Sample numbers are PRJNA610666, SRS6351352, and SAMN14309518, respectively.
